# Discrimination of benign and malignant non‐mass breast lesions using ultrasound radiomics with machine learning models

**DOI:** 10.1002/acm2.70319

**Published:** 2025-12-29

**Authors:** Chunming Shi, Huajun He, Bin Chen, Jiajia Lu, Qi Xu, Kai Zhao, Xiaoqing Yang

**Affiliations:** ^1^ Department of Ultrasound the First People's Hospital of Xiaoshan District Hangzhou China

**Keywords:** benign lesions, machine learning, non‐mass breast lesions, radiomics

## Abstract

**Aims:**

This study aims to enhance the preoperative diagnosis of non‐mass breast lesions (NMLs) by validating radiomics‐based machine learning models and assessing their performance alone and in combination with clinical ultrasound features to distinguish benign from malignant lesions.

**Methods:**

A total of 123 NMLs from 119 patients with confirmed pathology were analyzed. Patients were split into a training cohort (*n* = 98) and a validation cohort (*n* = 25). From each ultrasound image, 1558 radiomics features were extracted. After dimensionality reduction and feature selection, 10 key features were retained. Predictive models were developed using logistic regression (LR), linear regression, support vector machine (SVM), random forests, Extremely Randomized Trees (Extra Trees), and Light Gradient Boosting Machine (LightGBM). A clinical model was built using LR based on ultrasound findings such as calcification, high resistance index, and axillary lymph node enlargement. A combined model incorporated both radiomics and clinical features. Model performance was evaluated using receiver operating characteristic (ROC) curves and decision curve analysis (DCA).

**Results:**

The LightGBM model achieved the highest radiomics‐only performance (AUC: 0.932 training; 0.867 validation). The clinical model achieved AUCs of 0.837 (training) and 0.790 (validation). The combined model outperformed both, with AUCs of 0.973 (training) and 0.933 (validation), and showed superior clinical benefit in DCA.

**Conclusions:**

Combining radiomics with clinical ultrasound data significantly improves diagnostic accuracy for NMLs, supporting better differentiation between benign and malignant lesions and aiding clinical decision‐making.

## 1 INTRODUCTION

Breast cancer is among the most common types of cancer and is still a significant source of mortality and disability among women.^1^, ^2^, ^3^ Breast lesion characterization is very crucial to facilitate early detection and prompt treatment to increase breast cancer patients’ survival rates.^4^, ^5^ Mass lesions (MLs) can be well diagnosed and classified using normal diagnostic equipment such as BI‐RADS; however, the non‐mass breast lesions (NMLs) present a major problem. Due to the lack of well‐margined masses or space‐occupied lesions, which are not evaluable by conventional BI‐RADS criteria, NMLs are defined. This has led to low specificity in diagnosing NMLs by ultrasound, which complicates clinical management and results in unnecessary or delayed biopsies.^6^ Breast ultrasound is a common imaging technique for identifying and staging breast lesions owing to its availability, no use of ionizing radiation, and real‐time imaging.^7^, ^8^ Nevertheless, the assessment of NMLs by conventional ultrasound is based on the radiologists’ interpretation, which has considerable inter‐observer variability and low sensitivity regarding benign/malignant differentiation.^9^ These complications have highlighted the importance of employing improved diagnostic techniques that would combine with and enhance the specificity and repeatability of ultrasound in detecting NMLs. The growing medical imaging field of radiomics enables scientists to extract substantial quantitative data from imaging information.^10^, ^11^

While radiomics extracts high‐throughput features concerning the tumor shape, texture, and intensity, it provides a finer characterization of lesions that could not be perceived by the naked eye. When acted in conjunction with machine learning algorithms, radiomics can provide a new paradigm in modeling and more accurate classification of lesions.^12^ These machine learning methods, including logistic regression (LR), support vector machine (SVM), random forest (RF), and gradient boosting methods, show effectiveness in working with extensive radiomics databases to generate accurate malignancy predictions.

In the context of mass breast lesions, the role of radiomics has been established, but the same cannot be said for NMLs.^13^ Due to specific imaging characteristics and the biological nature of NMLs, specific radiomics models need to be designed for this group of breast lesions. Moreover, traditional imaging phenotypes, including calcification, blood flow resistance index, and axillary lymph node enlargement, are also related to malignancy, which might provide complementary, valuable indicators when combined with radiomics features.^14^

It is noteworthy that the integration of radiomics features with clinical characteristics can develop an aggregated diagnostic model with better diagnostic performance than each separate model.^15^ The application gap between radiomics and machine learning methodology requires attention in the context of NMLs. To date, there is a dearth of literature that systematically incorporates radiomics features and clinical parameters to establish diagnostic models for NMLs. Moreover, there is a lack of information as to how different machine learning algorithms compare with each other in terms of performance in such a setting. These gaps in the literature suggest that a more robust study is required to assess and confirm the usability of radiomics in differentiating benign from malignant NMLs.

The present study intends to fill these gaps by proposing and establishing an ultrasound‐based radiomics signature for NMLs. Employing machine learning approaches and clinical characteristics again, the work aims at creating a strong prognostic model. By adding clinical variables with radiomics features, the study wants to enhance the specificity of diagnosis and make a more practical use of clinical data to assist in decision‐making about patients with non‐mass breast lesions.

## 2 MATERIALS AND METHODS

### 2.1 Patient population

**FIGURE 1:**
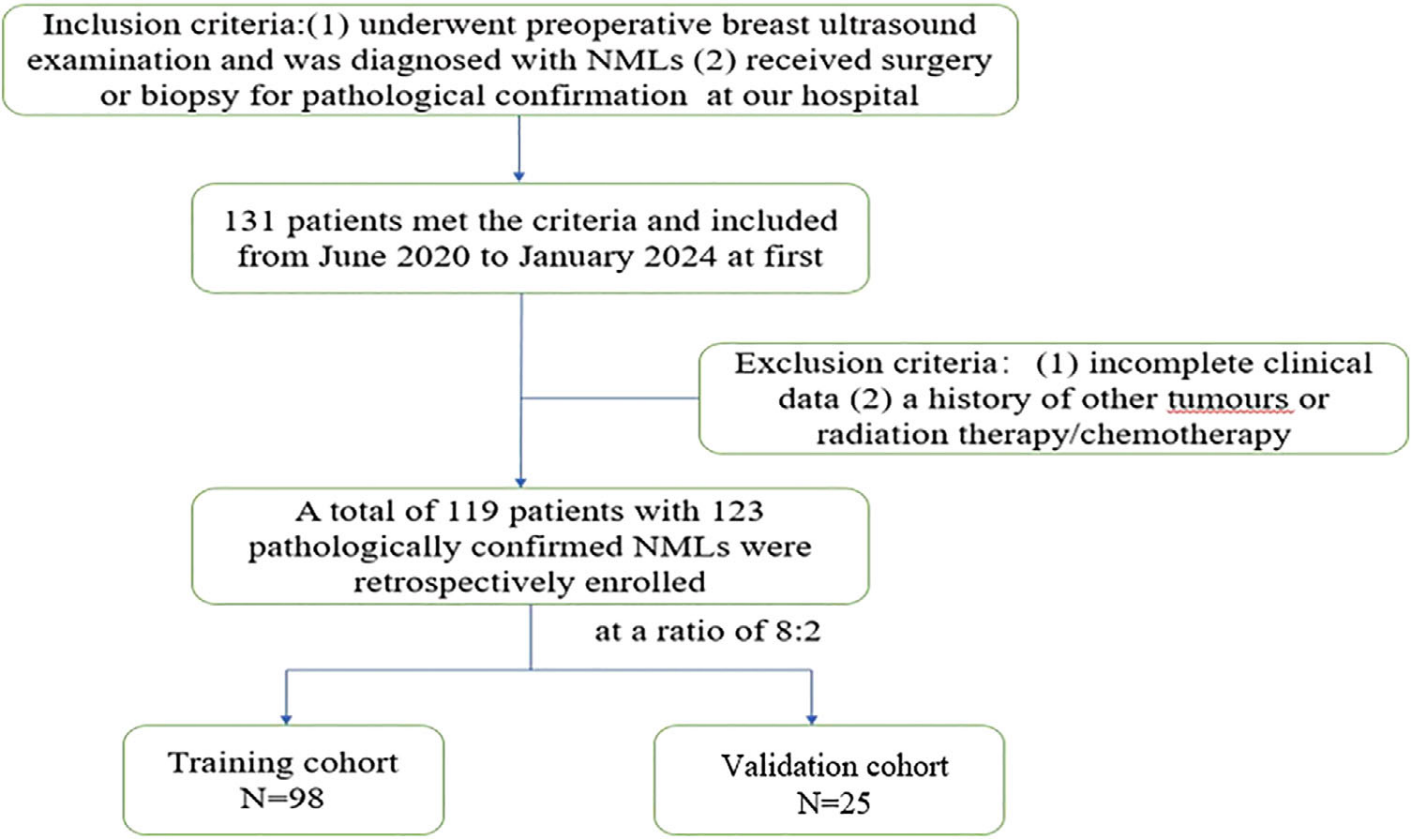
Flowchart of the patient selection process.

Between June 2020 and January 2024, our medical facility treated 119 patients with 123 lesions. Patient inclusion criteria were as follows: (1) had preoperative breast ultrasound examination and were diagnosed with NMLs, and (2) had surgery or biopsy for pathological confirmation at our hospital. Patient exclusion criteria were as follows: They can occur for (1) lack of complete clinical history or (2) the presence of other tumors or prior radiation therapy/chemotherapy. The lesion images were randomly allocated to a training and validation cohort at an 8:2 ratio; all data are presented in detail in Figure 1. Details of the tumor types within the cohort of the study are shown in Table 1.

**TABLE 1:** Histological analysis of the breast tumors in the study patients.

Histological type	Focus (*n*)	%
Benign tumors	67	54.5
Mammary adenopathy	32	26.0
Mastitis	16	13.0
Fibroadenoma	10	8.2
Intraductal papilloma	8	6.5
Sclerosing adenopathy	1	0.8
Malignant tumors	56	45.5
Invasive ductal carcinoma	31	25.2
Ductal carcinoma in situ	18	14.7
Intraductal papillary carcinomas	2	1.6
Lobular carcinoma in situ	3	2.4
Invasive lobular carcinoma	2	1.6

### 2.2 Image acquisition

All images were obtained using the SIEMENS ACUSON Sequoia with the 18L6 high‐frequency probe. Patients were supine with the area to be examined exposed and scanned in both medial and lateral projections. They were also evaluated for non‐mass lesions in multiple planes. One maximum cut‐face image per lesion was then kept for analysis.

### 2.3 Ultrasound images and clinical data analysis

Medical images and clinical data came from our institution's regular clinical practice and the Picture Archiving and Communication System (PACS). The retrospective evaluation included clinical parameters from patient records that combined surgical patient age with pathology outcome and sonographic findings of the lesion (internal echo pattern and calcification, along with RI > 0.7,^7^ axillary LN). Two radiologists evaluated the ultrasound images obtained in their combined 5+ years and 20+ years of professional experience.

If two radiologists had similar outcomes, the evaluation results were documented. In the case of disparity, another sonographer with more than 30 years of experience was sought, and a conclusion was made. Before the evaluation, all three radiologists had no information about the pathological results.

### 2.4 Image segmentation

**FIGURE 2:**
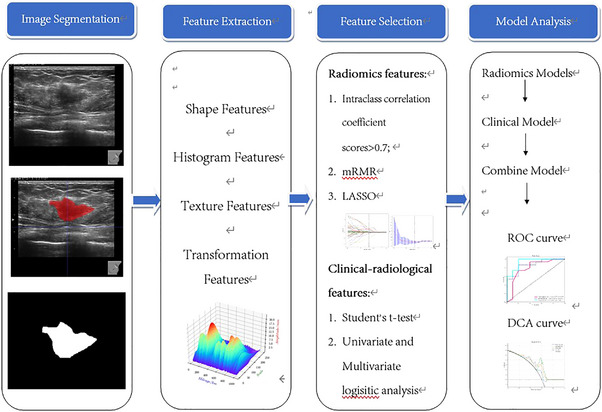
Workflow chart of the study.

Two experienced radiologists (5 and 20 years, respectively) manually segmented the regions of interest (ROIs) from DICOM ultrasound images using ITK‐SNAP. To ensure segmentation consistency, both readers independently segmented all lesions. The resulting segmentations were compared visually. For most cases, differences were minimal. However, when significant discrepancies occurred, a third expert radiologist with over 30 years of experience reviewed the case and reached consensus with the team. While this qualitative validation ensured consistency, we acknowledge that quantitative metrics like the Dice Similarity Coefficient (DSC) or intraclass correlation coefficient (ICC) were not calculated in this preliminary study. We aim to include inter‐observer agreement statistics in future multi‐center validations. This study utilized the workflow chart illustrated in Figure 2 below.

### 2.5 Radiomics feature extraction and selection

Three groups of radiomics features could be extracted from these ROI areas: Multiple characteristics encompass forms together with the degree of filling, textures, and shapes. Tumor shape properties determine the spatial dimensions of tissue morphology, which constitutes geometric features. Intensity features describe first‐order statistical distribution patterns of voxel intensities inside tumors. Estimating intensity patterns across different spatial scales occurs through texture feature analysis. The acquisition of textured features involved four different matrix types: gray level co‐occurrence matrix (GLCM), gray level run length matrix (GLRLM), gray level size region matrix (GLSZM), and neighborhood gray level difference matrix (NGTDM). The software package used in this study is Pyradiomics. This open‐source Python toolkit focuses on extracting high‐throughput quantitative features (called Radiomics features) from medical images, which are widely used to assist disease diagnosis, prognosis assessment, and treatment analysis. Although PyRadiomics partially meets the IBSI standard, its core advantage lies in its flexibility and configurability, which can meet different standards (such as IBSI, QIBA, etc.) through parameter adjustment.

The statistical significance of *t*‐tests was conducted on all the extracted radiomics features, and only features with *p*‐values less than 0.05 were used in further analysis. Spearman correlation coefficients were used for highly correlated features, with all pairwise feature correlation coefficients >0.7 retained in only one feature. The greatest extent of feature description was retained by applying the greedy recursive deletion strategy to filter features. In this strategy, most of the features in the current set were removed each time, most of the time. Finally, 46 were selected as the final feature set.

**FIGURE 3:**
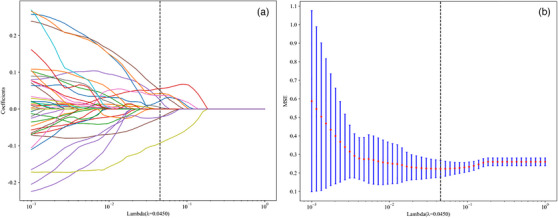
Feature selection using the LASSO regression model. (a) The tuning parameter (λ) selection in the LASSO model utilizes 10‐fold cross‐validation via minimum criteria. The vertical lines indicate the optimal value of the LASSO tuning parameter (λ). (b) LASSO coefficient profile plot with different log (λ). The vertical dashed lines represent 10 radiomics features with nonzero coefficients selected with the optimal λ value. LASSO, least absolute shrinkage and selection operator.

Using data from these studies, a LASSO regression model was employed to develop the signature. As the adjustment weight λ increases, all the regression coefficients are shrunk to 0 with the LASSO model; moreover, it sets several coefficients of unrelated features to be exactly 0. The optimal λ was identified by minimizing the cross‐validation sum from 10 to 1, and the value of λ chosen gave the minimal value of cross‐validation error. The remaining 10 non‐zero coefficient features were used as final imaging features for regression model construction (Figure 3).

### 2.6 Radiomics signature models

After the LASSO feature screening produced its last identified features, these elements served as the foundation for building the radiomics model. For this purpose, we have chosen different sets of machine learning algorithms to train the model: The team built predictive models using LR alongside SVM and RF, Extremely Randomized Trees (Extra Trees), and Light Gradient Boosting Machine (LightGBM). We conducted five‐fold cross‐validation to develop the final radiomics signature since it met performance standards while demonstrating high‐reliability metrics.

### 2.7 Construction and validation of the clinical and combined models

A similar approach was employed to develop the clinical model as was used for constructing the radiomics models. The researchers assessed clinical ultrasound features, which demonstrated statistical significance during both univariate and multivariate LR analyses (*p* < 0.05) to establish independent risk factors for malignancy in NMLs for their diagnostic model development. Radiomics signatures joined with selected clinical risk factors generated the integrated diagnostic model. The assessment of the combined model's quality and fair comparison used calibration curves for model fit analysis, which guided the creation of machine learning components. The diagnostic accuracy of the proposed model was evaluated through receiver operating characteristic (ROC) curve analysis combined with calibration curves and Hosmer‐Lemeshow tests to determine calibration efficiency and overall model reliability. Decision curve analysis (DCA) evaluated the practical advantages of decision‐making features for the clinical and radiomics models, along with the combined model.

### 2.8 Ethical considerations

The research received institutional ethics approval to proceed. The research team gathered informed consent from every participant who received complete information about the study's objectives and research approaches, as well as potential study risks. The study protected patient data confidentiality through strict measures while following the Declaration of Helsinki standards for all procedures.

### 2.9 Statistical analysis

Relevant software packages generated the analysis results of this study. When working with two normally distributed quantitative variables, researchers employed the *t*‐test, yet the *U*‐test replaced the t‐test when variables displayed non‐normal distribution patterns. The researchers used the chi‐square test together with Fisher's exact test to analyze descriptive variables for classification. A multivariable LR analysis with a forward selection method sought independent variables through a significance assessment at a *p* < 0.05 threshold.

## 3 RESULTS

### 3.1 Characteristics of the study patients

A total of 119 patients participated in the study, which included 123 patients diagnosed with non‐mass lesions (NMLs). A random split assigned 98 lesions to the training set, while the remaining 25 lesions went to the validation set. The two groups were matched as regards demographic and clinical variables at baseline, and no differences were found between the two groups that could be significant at the *p* < 0.05 level. As shown in Table 2, this group of patients had their baseline characteristics documented in detail.

**TABLE 2:** Patient baseline characteristics in the training and validation cohorts.

Variable	Training cohort (*n* = 98)			Validation cohort (*n* = 25)		
	Benign	Malignant		Benign	Malignant	
	(*n* = 57)	(*n* = 41)	*p*‐value	(*n* = 10)	(*n* = 15)	*p*‐Value
Age	46.02 ± 14.85	53.07 ± 11.82	0.013^*^	47.00 ± 14.91	56.93 ± 11.37	0.071
Internal			0.812			0.934
Echo uniform	35 (61.40%)	27 (65.85%)		5 (50.00%)	9 (60.00%)	
Uneven	22 (38.60%)	14 (34.15%)		5 (50.00%)	6 (40.00%)	
Rear echo			0.914			0.785
Attenuation No	47 (82.46%)	35 (85.37%)		8 (80.00%)	10 (66.67%)	
Yes	10 (17.54%)	6 (14.63%)		2 (20.00%)	5 (33.33%)	
Calcification			0.004^*^			0.084
No	45 (78.95%)	20 (48.78%)		7 (70.00%)	4 (26.67%)	
Yes	12 (21.05%)	21 (51.22%)		3 (30.00%)	11 (73.33%)	
Blood flow			<0.001^*^			0.679
Resistance No^a^	27 (47.37%)	9 (21.95%)		4 (40.00%)	4 (26.67%)	
Index Low	25 (43.86%)	15 (36.59%)		3 (30.00%)	4 (26.67%)	
High	5 (8.77%)	17 (41.46%)		3 (30.00%)	7 (46.67%)	
Enlarged			<0.001^*^			0.540
Axillary No	51 (89.46%)	20 (48.72%)		8 (80.00%)	9 (60.00%)	
Lymph nodes	6 (10.53%)	21 (51.22%)		2 (20.00%)	6 (40.00%)	

*Note*: Numerical data are presented as mean ± standard deviation. Categorical data are expressed as numbers (%).

^a^ Represents NMLs that have no obvious blood flow signal.

* *p* < 0.05.

Using univariate LR analysis, calcification, high blood flow resistance index, and axillary lymph node enlargement were found to have a statistical probability of being malignant for NML (Table 3). These three characteristics were then used to build the clinical predictive model for malignancy.

**TABLE 3:** Univariate and multivariate logistic regression analyses of the clinical features.

Variable	Univariate analysis		Multivariate analysis	
	OR (95% CI)	*p*‐Value	OR (95% CI)	*p*‐Value
Age	1.009 (1.003, 1.015)	0.013^*^	1.005 (1.000, 1.010)	0.106
Internal echo	0.954 (0.803, 1.135)	0.656	–	–
Rear echo attenuation	0.950 (0.757, 1.190)	0.704	–	–
Calcification	1.389 (1.175, 1.644)	0.002^*^	1.237 (1.065, 1.436)	0.020^*^
Blood flow resistance index	1.281 (1.156, 1.419)	<0.001^*^	1.161 (1.054, 1.279)	0.012^*^
Enlarged axillary lymph nodes	1.642 (1.390, 1.941)	<0.001^*^	1.474 (1.256, 1.730)	<0.001^*^

Abbreviations: CI, confidence interval; OR, odds ratio.

* *p* < 0.05.

### 3.2 Radiomics signature models and performance

One region of interest generated 1558 distinct variables in the radiomics analysis. The selection process identified 10 non‐zero coefficients, which were used for model development (Table 5). The LASSO regression model presented its feature selection method visually through Figure 3 to obtain optimal results. The suggested tuning parameter (λ) selection for feature selection appears in Figure 3a and Figure 3b and displays the coefficient profile plot of the selected features.

**FIGURE 4:**
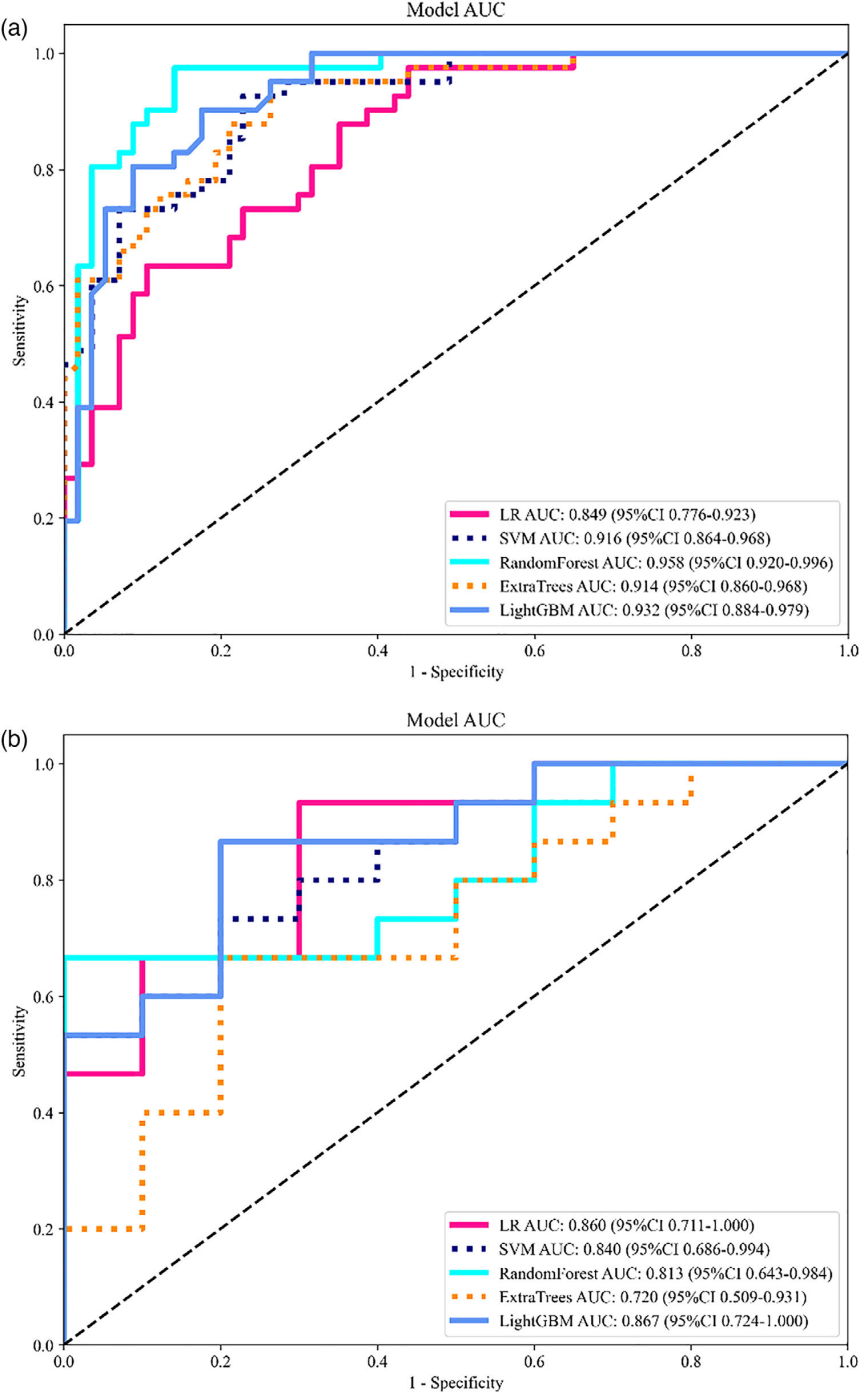
ROC curves of the LR, SVM, RF, Extra Trees, and LightGBM models in the training (a) and validation (b) cohorts. LR, logistic regression; RF, random forest; ROC, receiver operating characteristic; SVM, support vector machine.

The diagnostic capabilities of five machine learning models were evaluated by analyzing training and validation cohort data. Five machine learning models participated in the analysis: SVM and LR functioned with RF as well as Extra Trees, while operating alongside a LightGBM. RF emerged as the premier diagnostic tool in the training cohort by producing an AUC score of 0.958 with a 0.898 accuracy rate, 0.951 sensitivity, 0.860 specificity, 0.830 positive predictive value (PPV), and a negative predictive value (NPV) of 0.961 (Table 4). The RF model showed signs of overfitting because Figure 4 shows its performance metrics decreasing in the validation cohort. When the AUC value range of the model is too broad, it indicates that the model performance is unstable, which may be caused by data inconsistency, feature noise, model overfitting, or method defects. After analysis and discussion, this research group came up with some follow‐up solutions: (1) It may be due to the influence of feature noise. Since the ROI delineation of the ultrasound image was manually delineated, although senior and junior physicians completed it, it is difficult to avoid cases of inaccurate delineation. (2) The sample size is small. In subsequent work, we will seek multi‐center cooperation to increase the sample size and improve the stability of the model. (3) Through parameter tuning, such as adjusting the model complexity and regularization parameters, prevent overfitting, and follow standardized processes (such as IBSI) to improve the comparability and repeatability of the results. A combined model was constructed using LightGBM and clinical features.

**TABLE 4:** Results of the radiomics feature selection.

Model	AUC (95% CI)	Accuracy	Sensitivity	Specificity	PPV	NPV
LR Training	0.849 (0.776, 0.923)	0.724	0.951	0.561	0.609	0.941
Test	0.860 (0.711, 1.00)	0.800	0.867	0.700	0.812	0.778
SVM Training	0.916 (0.864, 0.969)	0.827	0.902	0.772	0.740	0.902
Test	0.840 (0.686, 0.994)	0.680	0.467	1.000	1.000	0.556
RF Training	0.958 (0.920, 0.996)	0.898	0.951	0.860	0.830	0.961
Test	0.813 (0.643, 0.984)	0.760	0.600	1.000	1.000	0.625
Extra Training Trees	0.914 (0.860, 0.968)	0.816	0.927	0.737	0.717	0.933
Test	0.720 (0.509, 0.931)	0.680	0.600	0.800	0.818	0.571
LightGBM Training	0.932 (0.884, 0.979)	0.847	0.878	0.825	0.783	0.904
Test	0.867 (0.724, 1.000)	0.800	0.800	0.800	0.857	0.727
Clinical Training	0.837 (0.753, 0.921)	0.786	0.585	0.930	0.857	0.757
Test	0.790 (0.583, 0.997)	0.760	0.867	0.600	0.765	0.750
Combined* Training	0.973 (0.949, 0.998)	0.908	0.976	0.860	0.833	0.980
Test	0.933 (0.833, 0.990)	0.880	0.933	0.800	0.875	0.889

Abbreviations: LR, logistic regression; NPV, negative predictive value; PPV, positive predictive value; RF, random forest; SVM, support vector machine.

The LightGBM model demonstrated the best performance metrics during testing of the validation cohort with an AUC of 0.867 and accuracy of 0.800, alongside sensitivity of 0.800, specificity of 0.800, PPV of 0.857, and NPV of 0.727 (Table 5). LightGBM maintained equivalent performances between training and validation sets, which qualified it for further model evaluation. Among the 10 selected radiomic features, wavelet‐derived first‐order features (Median, Kurtosis, Range) and texture‐based GLCM/NGTDM features consistently appeared across multiple machine learning models, suggesting they provide robust discrimination of NML malignancy. This stability across models indicates that certain texture and intensity descriptors may capture fundamental biological differences between benign and malignant NMLs.

**TABLE 5:** Diagnostic performance of the different machine learning models for predicting benign and malignant NMLs.

ID	Radiomics feature
1	exponential_glcm_Idn
2	exponential_glrlm_RunEntropy
3	exponential_glszm_SmallAreaLowGrayLevelEmphasis
4	squareroot_glcm_InverseVariance
5	squareroot_ngtdm_Complexity
6	wavelet_HHL_firstorder_Median
7	wavelet_LLL_firstorder_Kurtosis
8	wavelet_LLL_firstorder_Range
9	wavelet_LLL_glcm_Idn
10	wavelet_LLL_ngtdm_Coarseness

*Note*: Model was constructed using LightGBM and clinical models.

Abbreviations: AUC, area under the curve; ET, extremely randomized trees; LightGBM, light gradient boosting machine; LR, logistic regression; NPV, negative predictive value; PPV, positive predictive value; RF, random forest; SVM, support vector machine.

### 3.3 Construction and validation of the combined model

The combined diagnostic model included three selected clinical indicators (calcification and high blood flow RI, and axillary LN enlargement) along with ten selected radiomics features. The combined model received an independent evaluation against the individual clinical and radiomics models.

**FIGURE 5:**
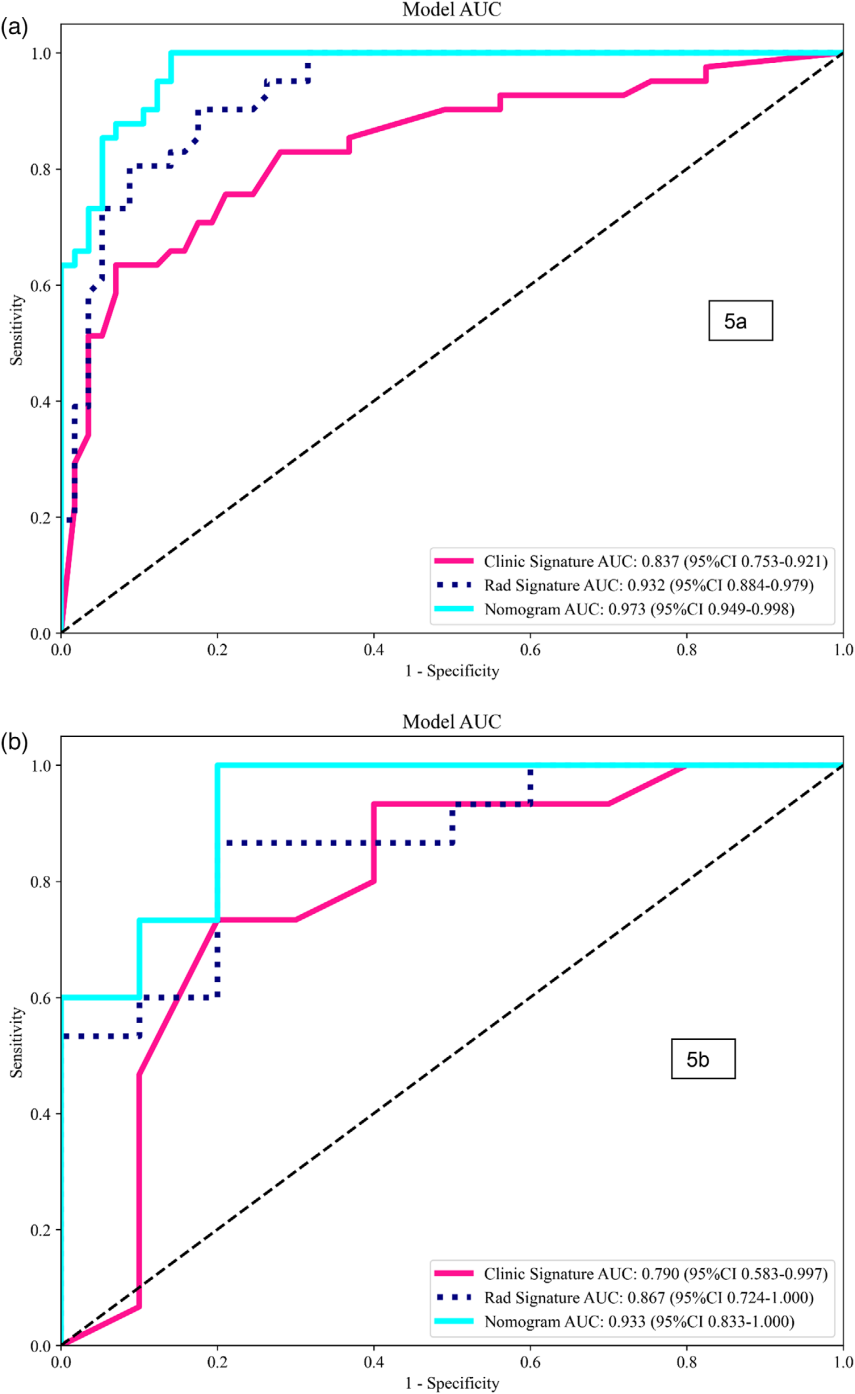
ROC curves of the radiomics, clinical, and combined models when predicting malignancy in the training (a) and validation cohorts (b). ROC, receiver operating characteristic.

The results show that the integrated model delivered superior diagnostic accuracy across both training and validation datasets compared to individual models. The combined model applied to the training data achieved an AUC of 0.973, together with an accuracy of 90.8%, a sensitivity of 97.6%, specificity of 86.0%, a PPV of 83.3%, and an NPV of 98.0%. The evaluation of the test data showed consistent results, having an AUC value of 0.933, together with an accuracy rate of 0.880, sensitivity of 0.933, specificity of 0.800, PPV of 0.875, and NPV of 0.889 (Figure 5).

### 3.4 Decision curve analysis

**FIGURE 6:**
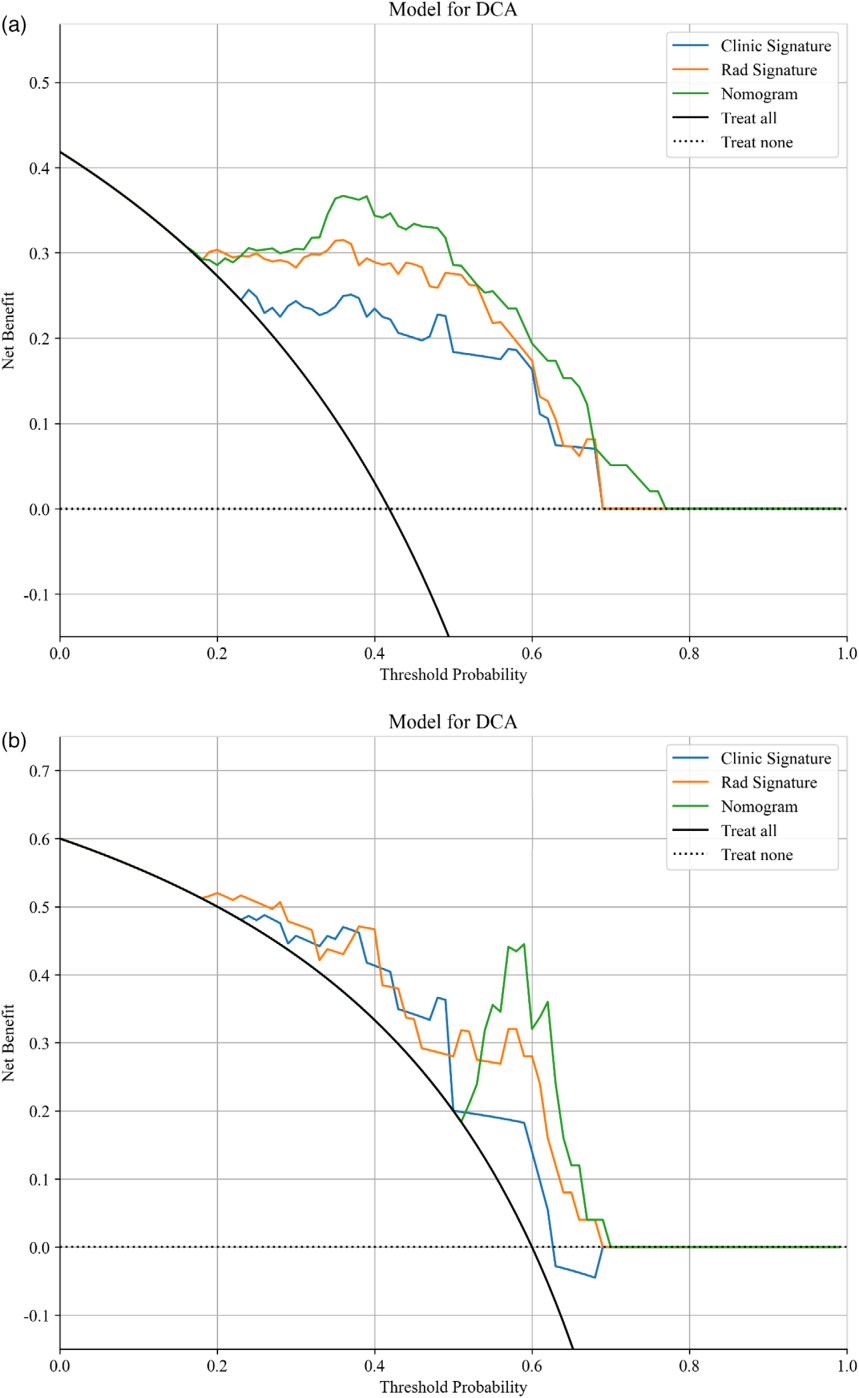
DCA of the three models for classifying NMLs in the training (a) and validation (b) cohorts. The graphs show that the combined model provides the greatest net benefit in the two cohorts. The graphs show that the combined model provides the greatest net benefit in the two cohorts. DCA, decision curve analysis.

DCA served as the final method to measure the clinical usefulness of the developed models. The proposed combined model demonstrated superior performance than individual models in achieving optimal treatment outcomes by distinguishing between benign and malignant NMLs over training and evaluation datasets, according to Figure 6. The model reveals a potential clinical implementation that supports both clinical decision processes and diagnostic accuracy.

## 4 DISCUSSION

Of all the screening tests for breast abnormalities, ultrasonography is the most common.^16^ However, improvement in high‐frequency ultrasound technology has enhanced the detection of NML, and it takes about 5.3%–9.2% of total breast diseases according to many studies.^17^, ^18^, ^19^ NMLs are described as structures with ill‐defined margins and without occupying space, and this might result in the missing of disorders while performing ultrasound scans. Secondly, it has been identified that some neoplastic and non‐neoplastic features are similar, which has led to low diagnostic accuracy of NMLs.^20^ NMLs have been categorized by many investigators worldwide according to their ultrasonic characteristics to improve the accuracy of ultrasound diagnosis.^21^, ^22^, ^23^

Earlier research on benign non‐mass lesions has revealed that fibrocystic changes in the breast are the most common histological type.^22^ On the other hand, malignant NMLs might comprise intraductal, invasive lobular, and invasive ductal carcinomas, and similar results were observed in the present study (Table 1). In the present study, 123 NMLs were included, and the distribution of benign and malignant lesions, which constituted 54.5% and 45.5%, respectively, matched with the findings of Park et al.^24^ The multiple NMLs show low rates of benign and malignant disease occurrence. The scientific community investigates current ultrasonic diagnostic techniques that combine contrast‐enhanced ultrasound with shear wave electrography to enhance the identification of NML.^25^, ^26^ Nevertheless, there has been a lack of studies on radiomics techniques for diagnosing NML.

Radiomics is a noninvasive approach that employs AI techniques to generate high‐throughput factors from unaltered digital imaging for evaluation to understand the condition, its evolution, and its response to different treatments.^27^, ^28^ Comparing five different radiomics models, we identified that the LightGBM model is the most stable and efficient one among the five proposed models. LightGBM is an efficient and scalable machine learning algorithm that belongs to the gradient‐boosting decision trees. In this study, the LightGBM model performed well in the training and validation sets (AUC, training cohort: 0.932; validation cohort: 0.867). The RF model achieved appropriate diagnostic value in the training dataset yet displayed significant diagnostic inaccuracy for the validation dataset, which suggests possible overfitting.^29^ Importantly, our feature analysis revealed that wavelet‐based first‐order statistics and texture‐derived measures were consistently retained across multiple machine learning algorithms. These features are biologically plausible, as they quantify lesion heterogeneity and textural complexity, which have been associated with malignancy in previous ultrasound and MRI radiomics studies.^13^, ^19^, ^22^ This strengthens the clinical interpretability of our model and highlights potential biomarkers that may be transferable across imaging modalities.

When developing the clinical feature model in the course of the study, calcification, high blood flow resistance index, and enlarged axillary lymph nodes have been recognized as effective ultrasound signs to predict malignancy in NMLs, as Zhi Li Wang et al and Kai‐Hsiung Koa et al also mentioned.^22^, ^23^ Most specifically, the authors determined that microcalcifications in a lesion were significantly related to malignant NMLs.

The radiomics features derived through LightGBM analysis combined with clinical parameters resulted in elevated diagnostic accuracy, yielding a training cohort AUC of 0.973 and a validation cohort AUC of 0.933. By combining these measurement techniques, the detection capability improved beyond what each method could achieve alone, exceeding individual radiomics and clinical model performance.

The current study was a retrospective, single‐center study, so selection biases may have been included in the study design. This might have been due to subjectivity arising from the manual definition of the segmented boundaries in this study; this should be amended in subsequent studies by automatically segmenting the ROIs. This study used only one ultrasound image per lesion, which might have restricted the quantity of radiomics information. Such future perspectives should consider utilizing them in videos to extract frames for re‐segmentation of the same to improve the model with larger samples.

The sample size of this study is small because breast lesions are mainly manifested as mass breast lesions (MLs), while non‐mass breast lesions (NMLs) account for a small proportion of breast lesions, accounting for only a small part (5.3‐9.2%). This study only retrospectively followed up patients from 2020 to 2024. If it is traced back further, it will lead to inconsistencies in the models of ultrasound examination equipment, which will affect the research results and make them unreliable. Therefore, the sample size of this study is the maximum sample size that can be followed up. In subsequent work, we will continue to increase the sample size and improve the stability of the model.

The lack of external validation is mainly because this is a preliminary study based on a single center. Obtaining an external validation set is difficult due to medical ethics and other reasons. In subsequent work, we will seek multi‐center cooperation to prove the model's universality through an external validation set.

## 5 CLINICAL IMPLICATIONS AND STUDY LIMITATIONS

This study highlights the diagnostic potential of combining radiomics features with clinical ultrasound parameters to improve the differentiation of benign and malignant non‐mass breast lesions (NMLs). Given the diagnostic ambiguity of NMLs in conventional ultrasound and their exclusion from BI‐RADS until recently, our model offers an evidence‐based tool that could enhance diagnostic confidence, reduce unnecessary biopsies, and support more individualized patient management. The integration of interpretable clinical features—such as calcification and axillary lymph node status—also facilitates adoption in routine practice.

Despite its strengths, the study has inherent limitations. As a single‐center retrospective analysis, its findings may be influenced by institutional bias and limited sample diversity. The modest cohort size, although maximized for this rare lesion subtype, may affect the generalizability of results. Additionally, manual segmentation of ultrasound images, while cross‐validated among experienced radiologists, introduces variability that automated methods could mitigate in future work. The absence of external validation further constrains the robustness of the model. Moving forward, multi‐center collaborations, integration of elastography or contrast‐enhanced modalities, and video‐based radiomics could strengthen model performance and clinical translation.

We acknowledge that our study applies established radiomics and machine learning techniques without introducing methodological innovations; this represents a limitation. Nevertheless, our work contributes to the field by applying these techniques to the underexplored area of non‐mass breast lesions and highlighting the challenges and future directions required to enhance diagnostic accuracy in this context.

Previous studies on radiomics in breast ultrasound have primarily focused on mass lesions rather than NMLs.^13^, ^17^, ^22^ Our findings are consistent with Li et al. and Ko et al., who reported that texture‐based radiomic features enhance the discrimination of malignant lesions. However, unlike prior studies, our work integrates both radiomic and clinical features in NMLs, highlighting a complementary diagnostic value that has been underexplored in the literature.

## 6 CONCLUSION

The current study established and tested the combined model for diagnosing benign and malignant NMLs employing ultrasonography image features. In this work, the complementary model incorporating LightGBM‐based radiomics features and clinical models had the optimal diagnostic accuracy and impact in discriminating benign from malignant NMLs in both training and validation sets, offering a novel method for diagnosing and managing NMLs.

## AUTHOR CONTRIBUTIONS

All authors contributed substantially to the work and approved the final version of the manuscript. **Chunming Shi** contributed to the study conception and design, data analysis, and manuscript drafting. **Huajun He** participated in radiomics feature extraction and image processing. **Bin Chen** contributed to clinical data interpretation and statistical analysis. **Jiajia Lu** assisted with machine learning model development and validation. **Kai Zhao** contributed to the critical revision of the manuscript and methodological support. **Xiaoqing Yang** supervised the study, provided expert guidance, and approved the final manuscript for submission.

## CONFLICT OF INTEREST STATEMENT

The authors declare no conflicts of interest.
